# Coverage for evidence-based cancer survivorship care services

**DOI:** 10.1007/s00520-024-08359-9

**Published:** 2024-02-17

**Authors:** Anne H. Blaes, Maysa M. Abu-Khalaf, Catherine M. Bender, Susan F. Dent, Chunkit Fung, Sophia K. Smith, Samantha Watson, Sweatha Katta, Janette K. Merrill, Shawna V. Hudson

**Affiliations:** 1 University of Minnesota, Minneapolis, MN USA; 2 Thomas Jefferson University, Philadelphia, PA USA; 3 University of Pittsburgh, Pittsburgh, PA USA; 4 Duke University Durham, Durham, NC USA; 5 University of Rochester, Rochester, NY USA; 6 Samfund, Boston, MA USA; 7 American Society of Clinical Oncology, Alexandria, VA USA; 8 Rutgers University, Rutgers, NJ USA

**Keywords:** Cancer, Survivors, Survivorship care services

## Abstract

**Purpose:**

The American Society of Clinical Oncology Cancer Survivorship Committee established a task force to determine which survivorship care services were being denied by public and private payers for coverage and reimbursement.

**Methods:**

A quantitative survey instrument was developed to determine the clinical practice-reported rates of coverage denials for evidence-based cancer survivorship care services. Additionally, qualitative interviews were conducted to understand whether coverage denials were based on payer policies, cost-sharing, or prior authorization.

**Results:**

Of 122 respondents from 50 states, respondents reported that coverage denials were common (“always,” “most of the time,” or “some of the time”) for maintenance therapies, screening for new primary cancers or cancer recurrence. Respondents reported that denials in coverage for maintenance therapies were highest for immunotherapy (41.74%) and maintenance chemotherapy (40.17%). Coverage denials for new primary cancer screenings were highest for Hodgkin lymphoma survivors needing a PET/CT scan (49.04%) and breast cancer survivors at a high risk of recurrence who needed an MRI (63.46%), respectively. More than half of survey respondents reported denials for symptom management and supportive care services. Fertility services, dental services when indicated, and mental health services were denied “always” or “most of the time” 23.1%, 22.5%, and 12.8%, respectively. Respondents reported they often had a process in place to automatically appeal denials for evidence-based services. The denial process, however, resulted in greater stress for the patient and provider.

**Conclusion:**

Our study demonstrates that additional advocacy with payers is needed to ensure that reimbursement policies are consistent with evidence-based survivorship care services.

**Supplementary Information:**

The online version contains supplementary material available at 10.1007/s00520-024-08359-9.

## Introduction

In 2006, the Institute of Medicine published a report titled “From Cancer Patient to Cancer Survivor.” This report describes the importance of providing comprehensive, coordinated care for cancer survivors [1]. The report highlighted the importance of preventing cancer recurrence and new cancers, providing surveillance for cancer recurrence and for medical and psychosocial late effects, managing the broad consequences of cancer and its treatment, and finally coordinating care among specialists and primary care providers. Subsequently, in 2013, the American Society of Clinical Oncology (ASCO) issued the statement *Achieving High-Quality Survivorship Care *[2] in which the Society committed to improving the care of cancer survivors. In addition to describing ASCO’s recent activity to prioritize a focus on survivorship care delivery, the statement offered recommendations for improving the care of cancer survivors, including the need for a specific focus on the development of clinical guidance to guide the management of survivors and ensure cancer survivors have access to appropriate services.

Despite advancements in reimbursement for survivorship services, anecdotal evidence suggests that patients are not able to access guideline concordant survivorship care services due to a lack of coverage by payers. To understand the extent of the issue and inform ASCO advocacy groups tasked with ensuring cancer survivors receive the full range of services dictated by evidence-based clinical guidelines, a task force of the ASCO Cancer Survivorship Committee conducted a descriptive study to determine which services are being denied by public and private payers for coverage and reimbursement. The project aimed to determine the clinical practice-reported rates of delay and denial of evidence-based, guideline concordant survivorship care services from payers, and to document the source of these delays. Utilization management policies such as step therapy limits to the number of visits allowed for supportive care and survivorship services and evaluation of cost-sharing programs that patients are often unable to afford were examined.

## Methods

### Quantitative data collection

A task force from the American Society of Clinical Oncology Cancer Survivorship Committee was established in 2018 to examine gaps in coverage for adjuvant therapies and survivorship care services. As a first step, the task force documented all guidelines available as of 2018 (Supplemental Table 1). At the time, evidence-based guidelines focused on breast, colorectal, gynecological, head and neck, lung, Hodgkin lymphoma, and prostate cancers [2–8]. Next, a quantitative survey instrument was developed to gather data on gaps in coverage for evidence-based cancer survivorship care services. While patients and providers use a variety of techniques to aid in survivorship recovery and care (i.e., integrative therapies), questions were limited to disease sites and supportive care for which clinical practice guidelines supported by best evidence of consensus are available.

The survey instrument was designed to capture those areas of care where deeper query and analysis are needed and to identify areas where coverage is inadequate. Questions asked participants to indicate their encounter with coverage denials for their patients for the following: maintenance therapies in the post-curative survivorship treatment period, screening for new primary cancers, screening for cancer recurrence, screening for second malignancies, and survivorship/supportive care services. Queries focused on breast, colorectal, gynecological, head and neck, lung, Hodgkin lymphoma, and prostate cancers, where specific guidelines were available. Available responses included the following: most of the time, some of the time, neutral/unsure, some of the time, never, or service not provided/offered. Further questions inquired which payors created the largest coverage barriers. Additional open-ended questions asked whether the number of allotted treatments was limited or whether cost sharing was too high, making it inaccessible for patients. Finally, respondents were asked whether they had a financial counselor or navigator and how this individual was utilized. The survey was reviewed and tested by members of ASCO’s Cancer Survivorship Committee; modifications in the survey were made based on these recommendations. The survey and administration plans were reviewed and approved by the ASCO Research Services Committee. Research was conducted in accordance with the American Society of Clinical Oncology Research Services Committee and the declaration of Helsinki. Given the anonymity of the survey, the survey and administration plans were reviewed and approved by the ASCO Research Services Committee; consent was waived.

### Identification of target cohort to survey

Task force members who advocate for survivorship care were personally familiar with the difficulties associated with obtaining coverage for survivorship services and recognized that access to these services is likely limited in other practices as well. The task force, therefore, aimed to identify a cohort familiar with survivorship care services to conduct their research. As an initial step in identifying sites to survey, the task force developed a list of survivorship clinics based on a previously compiled list of LIVESTRONG’s Centers of Survivorship Excellence and a survivorship care program directory previously developed for adolescents and young adults (AYA) from a volunteer. With a goal of obtaining representation from fifty states, a spectrum of patient populations (pediatric, AYA, and adult) and size (community, academic, and LIVESTRONG), this list was expanded. Providers, researchers, and/or advocates for survivorship care (current and former ASCO Survivorship Committee members, ASCO State Affiliates, ASCO members who volunteered for survivorship focus projects, and ASCO members who are practice administrators) from 97 clinics were identified. The survey was sent to these 553 ASCO members in February 2020. Given the development of the COVID-19 pandemic and demand on medical practices and providers, the survey was sent on one occasion only.

### Qualitative data collection

Eighty-seven individuals who completed the quantitative survey voluntarily agreed to be re-contacted for focused interviews. Aiming for diverse geographical representation, practice type, and provider type, a purposive sample of 24 respondents were invited for interviews between September and October 2020 to further understand the barriers to coverage of guideline concordant survivorship care services. The task force conducted six qualitative, semi-structured telephone interviews to explore the extent to which coverage barriers are experienced for guideline-concordant care, specific to the provider or clinic’s primary disease site or specialty, as well as to explore potential workflows for these denials (Supplemental Table 2). Two interviewers (AB and SH) conducted digitally recorded, semi-structured Zoom interviews that ranged in length from 30 to 60 min. At the completion of these interviews, common themes had been identified. Additional interviews were not pursued given the worsening of the COVID-19 pandemic.

### Analyses

Descriptive statistics were used to summarize survey responses and the proportion of respondents by demographic characteristics. Survey responses were categorized on a spectrum from “always” denied to “never” denied. In addition, respondents were asked if they provided or offered services; responses of “services not provided/offered” were excluded from the analysis as coverage denials are not possible in a clinical setting that does not offer the service. Analyses included data only for the categories of “always,” “most of the time,” “some of the time,” “never,” and “unsure.” Consistent with the Standards for Reporting Qualitative Research [9], an immersion/crystallization qualitative analysis [10] was conducted by SK and JM with supervision from SH and AB. Initially, the coders coded 3 interviews together to calibrate code definitions resolving disputes by consensus. Two coders (SK and JM) then coded the remaining data, with periodic meetings with the team to check for consistency. At the conclusion of data collection, the analysis team engaged in another round of immersion/crystallization with the coded data, resulting in a synthesis of facilitators and challenges most salient across the sites. The complete data set is available upon request.

## Results

### Respondent characteristics

The online survey received a total of 122 responses (22% response rate), with all 50 states and the District of Columbia represented (Table 1). Most respondents self-identified as clinicians (89%) with a terminal degree of MD or DO (87%), with nurse practitioners (7%), and PhDs (5%) representing most remaining respondents. When asked about type of payer mix within their clinic, a majority reported treating patients with Medicare/Medicaid (62%), followed by private or employer insurance (36%), Veterans Administration or other military coverage (4%), and uninsured (5%). Respondents reported that breast cancer was the most common cancer type treated (43%) followed by a general mix of cancers (37%) and prostate (25%), colorectal (23%), lung (22%), Hodgkin lymphoma (15%), head and neck (10%), and gynecologic cancers (9%).

**Table 1:** Demographics of respondents

Demographic	*N*	Percent of responders
Clinician (Y/N)		
Clinician Nonclinician	107 12	89.92% 10.08%
Terminal degree		
MD/DO PhD DNP NP RN PA	95 6 2 8 3 1	87.96% 5.56% 1.85% 7.41% 2.78% 0.93%
Role in practice		
Billing administrator Practice manager Clinician	1 11 105	0.87% 9.57% 91.30%
Payer mix percentage billed by your program/practice		
Medicare and Medicaid % Private or employer insurance % VA/other military Uninsured %		62% 36% 4% 5%
States and associated responses		
AZ, AR, CO, DE, DC, HI, ID, KS, KY, LA ME, MS, MO, NM, ND, OK, OR, RI, SD, UT, VT, VA, WA, WV, and WY	1	49%
CT, GA, IN, MN, MT, NE, NH, NJ, SC, TN, and WI	2	22%
AK, MA, NV, and PA	3	7%
AL, IL, IA, MI, and MC	4	10%
FL, MD, and OH	5	6%
TX	6	2%
CA	9	2%
NY	11	2%

### Frequency of coverage denials

Respondents indicated that denials in coverage for maintenance therapies were more common (always, most of the time, and some of the time) for immunotherapy (41.74%) and maintenance chemotherapy (40.17%) than for aromatase inhibitors (21.24%) and hormone therapies (20.00%) in the post-curative survivorship treatment period (Table 2). Denials occurring “always” or “most of the time” were rare; approximately 7% of respondents reported that denials occurred “always” or “most of the time” for maintenance immunotherapy.

**Table 2:** Survey questions and results on encounter with coverage denials

	Always	Most of the time	Some of the time	Neutral/unsure	Never	Service not offered/provided
Maintenance therapies						
Hormone therapy (e.g., tamoxifen and estrogen inhibitor) (*n* = 115)	0.00%	0.87%	19.13%	6.96%	55.65%	17.39%
Maintenance chemotherapy (*n* = 117)	0.85%	1.71%	37.61%	8.55%	30.77%	20.51%
Maintenance immunotherapy (*n* = 115)	1.74%	5.22%	34.78%	9.57%	24.35%	24.35%
Aromatase inhibitors (*n* = 113)	1.77%	1.77%	17.70%	7.08%	53.98%	17.70%
Screening for new primary cancers						
Breast cancer						
Annual mammogram (*n* = 98)	1.02%	2.04%	9.18%	3.06%	71.43%	13.27%
Annual gynecological assessment in post-menopausal women on SERMs (*n* = 109)	0.92%	2.75%	13.76%	14.68%	46.79%	21.10%
Colorectal cancer						
Annual imaging with CT scan (*n* = 105)	0.95%	6.67%	32.38%	8.57%	24.76%	26.67%
Lab work including CEA (*n* = 107)	0.00%	1.87%	24.30%	7.48%	42.06%	24.30%
Gynecologic cancer						
Physical examination for endometrial cancer (with full review of symptoms) (*n* = 100)	0.00%	2.00%	9.00%	13.00%	39.00%	37.00%
Testing of CA-125 levels in select patients who had an elevated level before treatment for ovarian cancer (*n* = 100)	0.00%	0.00%	23.00%	13.00%	30.00%	34.00%
CT scans of the chest for patients treated for uterine sarcomas (*n* = 100)	1.00%	3.00%	26.00%	17.00%	17.00%	36.00%
CT scans every 6 months for first 2 years after completion of curative treatment for ovarian cancer (*n* = 100)	1.00%	1.00%	23.00%	17.00%	22.00%	36.00%
Annual CT scan (beginning in year 3) after completion of curative treatment for ovarian cancer (*n* = 99)	1.01%	2.02%	21.21%	18.18%	21.21%	36.36%
Serial pelvic sonography for women who have undergone fertility-preserving surgery with or without tumor markers (*n* = 99)	1.01%	1.01%	10.10%	31.31%	14.14%	42.42%
Head and neck cancers						
Routine age and gender appropriate screening (*n* = 101)	0.00%	4.95%	11.88%	13.86%	34.65%	34.65%
Low-dose CT for high risk patients based on smoking history (*n* = 101)	0.00%	6.93%	33.66%	9.90%	20.79%	28.71%
Hodgkin lymphoma						
Laboratory studies, as indicated (e.g., CBC, platelets, ESR, and TSH) (*n* = 105)	0.00%	0.95%	10.48%	10.48%	50.48%	27.62%
CT scan with contrast as indicated (*n* = 104)	0.00%	4.81%	21.15%	8.65%	37.50%	27.88%
PET/CT if indicated (*n* = 104)	1.92%	10.58%	36.54%	8.65%	15.38%	26.92%
Lung cancer						
Low-dose CT scan (LDCT) (*n* = 103)	0.97%	3.88%	33.01%	10.68%	23.30%	28.16%
Screening for cancer recurrence or late effects of cancer treatment						
Breast cancer						
6-month post-treatment mammogram (*N* = 103)	0.00%	3.88%	22.33%	6.80%	47.57%	19.42%
Annual mammogram (*N* = 103)	0.00%	0.00%	8.74%	3.88%	69.90%	17.48%
MRI (patients meeting high risk) (*N* = 104)	1.92%	9.62%	51.92%	5.77%	13.46%	17.31%
Colorectal cancer						
Colorectal cancer screening (e.g., colonoscopy) (*n* = 102)	0.00%	0.98%	17.65%	12.75%	37.25%	31.37%
Screening for bladder cancer (for those treated for rectal cancer) (*n* = 102)	0.00%	1.96%	23.53%	20.59%	15.69%	38.24%
CEA testing (*n* = 100)	0.00%	3.00%	21.00%	7.00%	44.00%	25.00%
Annual abdominal and chest imagining with CT scan in first 3-year post-treatment (*n* = 102)	1.96%	3.92%	25.49%	9.80%	33.33%	25.49%
Surveillance colonoscopy (*n* = 102)	0.00%	0.98%	17.65%	7.84%	48.04%	25.49%
Gynecologic cancer						
Physical examination for endometrial cancer (with full review of symptoms) (*n* = 94)	0.00%	0.00%	5.32%	18.09%	41.49%	35.11%
Testing of CA-125 levels in select patients who had an elevated level before treatment for ovarian cancer (*n* = 94)	0.00%	3.19%	17.02%	14.89%	29.79%	35.11%
CT scans of the chest for patients treated for uterine sarcomas (*n* = 94)	1.06%	2.13%	22.34%	20.21%	20.21%	34.04%
CT scans every 6 months for first 2 years after completion of curative treatment for ovarian cancer (*n* = 93)	1.08%	2.15%	19.35%	18.28%	24.73%	34.41%
Annual CT scan (beginning in year 3) after completion of curative treatment for ovarian cancer (*n* = 93)	1.08%	3.23%	19.35%	16.13%	24.73%	35.48%
Serial pelvic sonography for women who have undergone fertility-preserving surgery with or without tumor markers (*n* = 93)	1.08%	3.23%	7.53%	23.66%	16.13%	48.39%
Head and neck cancer						
Physical examination every 1–3 months in first year post-treatment (*n* = 96)	0.00%	0.00%	9.38%	11.46%	53.13%	26.04%
Physical examination every 2–6 months in second year post-treatment (*n* = 96)	0.00%	2.08%	5.21%	12.50%	55.21%	25.00%
Physical examination every 4–8 months in years 3–5 post-treatment (*n* = 96)	0.00%	1.04%	9.38%	16.67%	47.92%	25.00%
Annual physical exam after 5 years (*n* = 96)	0.00%	2.08%	6.25%	16.67%	48.96%	26.04%
Hodgkin lymphoma						
Laboratory studies, as indicated (e.g., CBC, platelets, ESR, and TSH) (*n* = 101)	0.00%	0.99%	4.95%	10.89%	59.41%	23.76%
CT scan with contrast as indicated (*n* = 101)	0.00%	3.96%	18.81%	11.88%	41.58%	23.76%
PET/CT if indicated (*n* = 102)	4.90%	4.90%	37.25%	10.78%	18.63%	23.53%
Stress test/ECHO (*n* = 100)	0.00%	3.00%	21.00%	17.00%	31.00%	28.00%
Carotid ultrasound for those receiving neck radiation (*n* = 101)	0.00%	2.97%	17.82%	25.74%	22.77%	30.69%
Breast MRI in those with prior mantle radiation (*n* = 99)	4.04%	4.04%	36.36%	17.17%	14.14%	24.24%
Lung cancer						
Low-does CT screening (*n* = 99)	1.01%	6.06%	30.30%	10.10%	26.26%	26.26%
Prostate cancer						
Measure of serum PSA every 6–12 months within first 5 years (*n* = 98)	0.00%	2.04%	13.27%	9.18%	52.04%	23.47%
Annual measure of serum PSA after 5 years (*n* = 98)	0.00%	2.04%	9.18%	12.24%	53.06%	23.47%
Annual DRE (*n* = 98)	0.00%	2.04%	4.08%	12.24%	52.04%	29.59%

Respondents reported that coverage denials for screening for new primary cancers or cancer recurrence were more common for imaging exams than physician examinations, laboratory studies, or other screening procedures. Coverage denials for screening of new primary cancers were highest for PET/CT scan for Hodgkin lymphoma survivors (49.04%), low-dose CT for high-risk patients based on smoking history for head and neck cancer survivors (40.59%), annual CT scan for colorectal cancer survivors (39.90%), and low-dose CT scan for lung cancer survivors (37.86%). Coverage denials for screening of cancer recurrence were highest for MRI for patients with a high-risk of breast cancer recurrence (63.46%), PET/CT scan as indicated for Hodgkin lymphoma recurrence (47.05%), breast MRI in survivors of Hodgkin lymphoma at risk for breast cancer (44.44%), annual abdominal and chest CT scan in the first 3 years after treatment for colorectal recurrence (31.37%), and low-dose CT scan for lung cancer recurrence (37.37%). Overall, denials occurring “always” or “most of the time” were not common though they were highest for high risk breast cancer screening with breast MRI, and for PET/CT when indicated for Hodgkin lymphoma recurrence.

**Fig. 1:**
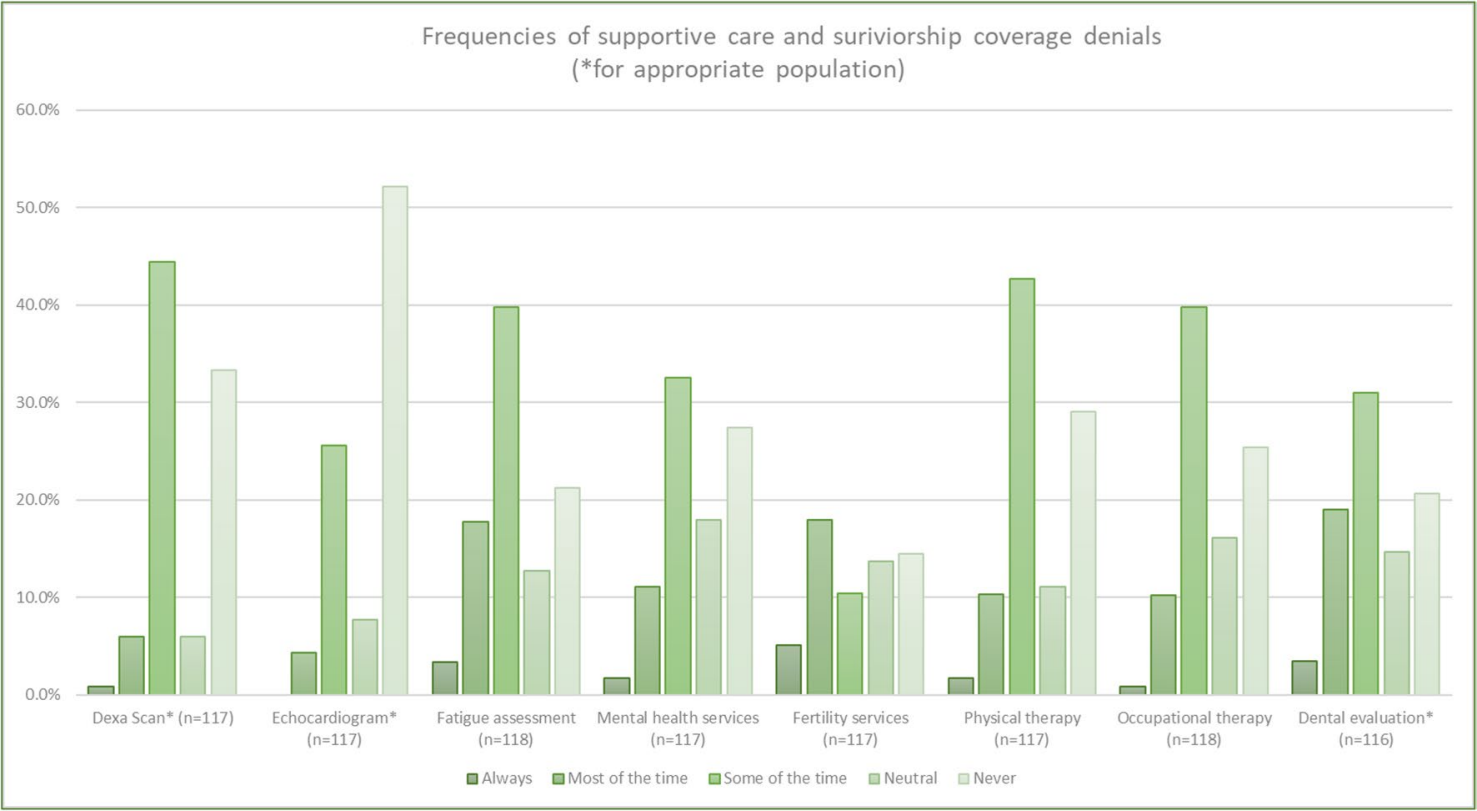
Frequencies of supportive care and survivorship coverage denials

More than half of the survey respondents reported denials for supportive care or symptom management services (Fig. 1), including fertility preservation (63.30%), fatigue assessment (61.00%), dental evaluations (53.50%), physical therapy (57.10%), bone density tests (51.30%), occupational therapy (50.90%), speech therapy (54.00%), and mental health services (45.30%). Fertility services, dental services when indicated, and mental health services were denied “always” or “most of the time” 23.1%, 22.5%, and 12.8%, respectively.

### Reasons for coverage denials

Though analyses of the responses did not clearly define an issue specific to a particular region or state, most respondents reported that private or employer-based insurance was most often the source of barriers (51%). To understand whether coverage denials were based on payer policies, cost-sharing, or prior authorization, qualitative interviews were conducted. Respondents indicated that lack of coverage was generally an issue of prior authorization, though most reported having a process in place to automatically appeal denials for evidence-based services (Table 3). The denial process, however, resulted in greater stress for the patient and contributed to provider burnout. The causes were found to be the same across sites and not unique to a single payer or region.

**Table 3:** Key findings from interviews

Insurance barriers *Number of survivors are increasing due to improved therapies, but payer policies are not expanding at the same rate*	• Stricter payer policies with varying requirements and financial burden falls on patient • Excessive documentation (beyond medical necessity) required by payers with citations from publications to show the justification • Site of care is an issue (what is done at home vs. center) • Regarding bone density denial—denied because the breast cancer patient does not have osteoporosis, but due to their treatment they are at a higher risk • Prior authorization and peer-to-peer common for survivorship services, but an added step and burden for provider • Private payers seem to have the most barriers
Patient barriers	• Patients are not aware that the practice is working behind the scenes to get the services covered and when the patient receives the denial, adds stress to an already stressful situation • Patient health insurance literacy plays a role
Solutions *Processes, systems, and models adopted by providers/practices*	• Practices are aware which services will be denied and have processes in place to get these services for their patients—but require a team which all interviewees (except VA interviewee) have in place • Patient navigators and social workers help reduce barriers including identifying the resources needed (i.e., gas cards for rural patients, explaining their insurance coverage, and identifying community resources) • Finding the right billing code can reduce barriers

## Discussion

Denial for survivorship care, particularly supportive care services, is frequent. While denial is common, it appears providers and programs often have a process to work through denials and prior authorization processes, regardless of payer type or region of the country. The difficulties posed by insurance policies with denials/deferrals inhibit the delivery of guideline concordant care; these challenges impose potential harm to patients, increase cost to the health care system, and impose undue burden and stress on clinicians and administrators with needs for letters and phone calls around appeal processes. There is a need to improve the advocacy supporting policies and practices that regulate re-imbursement for guideline concordant imaging studies in an effort to improve supportive care services for cancer survivors.

For over fifteen years since the Institute of Medicine released “Lost in Transition,” [1] it has been clear that cancer survivors have a unique trajectory after the completion of their cancer treatment. Based on genetics, age at receipt of cancer treatment, treatment exposures, and lifestyle, cancer survivors are at increased risk of cancer recurrence, secondary cancers, chronic health conditions, and impairments in quality of life as a consequence of aging, underlying risk factors, and prior cancer treatment [11]. Providing risk stratified guideline concordant care has been shown to improve the morbidity and mortality associated with secondary cancers in cancer survivors [12–14]. Similarly, improving access to supportive care services (i.e., cardiooncology, cancer rehabilitation, and psychooncology) has been demonstrated to help patients recover from the effects of their cancer and treatment-related impairments [15, 16]. Improving access to evidence-based survivorship care provides an opportunity to reduce the morbidity and mortality associated with a cancer diagnosis while also providing a platform for quality survivorship care [16].

Despite the development of guidelines and the potential for improved outcomes in cancer survivors in terms of reducing cancer recurrence and chronic diseases, adherence to guideline concordant survivorship care is low [17]. Barriers to adhering to guideline concordant care can include lack of patient knowledge, lack of provider knowledge surrounding the guidelines, increased risk of chronic disease, and lack of coverage to support these services [17–20]. The results of this study suggest denials for screening for recurrence or second cancers are common in breast, Hodgkin lymphoma, head and neck cancer, colorectal cancer, and lung cancer. Denials are also common for mental health services and supportive care tests and services (i.e., fertility and dental health services). Additionally, individuals with gaps in coverage are less likely to receive cancer prevention screening and treatment. One can hypothesize that denials in survivorship care services may result in poorer outcomes, given the fact that the services are ultimately not covered and then potentially not performed or performed in a delayed fashion. Working through the denial process can create delays in cancer screening and preventive healthcare screening; it also provides stress for the patient and provider. Prior studies suggest individuals with gaps in health insurance coverage present with more advanced cancers, are less likely to receive recommended cancer treatment, and have a worse overall survival.

Dealing with insurance denials and prior authorization requirements for treatments and procedures has been associated with physician and provider burnout. It also contributes to physician dissatisfaction. In a recent survey of 22,276 physicians of internal medicine specialties by Shanafelt and colleagues [21], approximately one in three providers had signs and symptoms of burnout measured by the Maslach Burnout Inventory scale. While there are many factors associated with burnout, encountering insurance denials and authorization requirements places increased stress on patients and providers. It also increases the economic burden on medical practices themselves [22]. Within our study, practices described a process to obtain insurance approval, although we did not explore the cost associated with these processes.

Future work would benefit from round table discussions with key stakeholders (patient advocates, providers, health care systems, payers, and policy makers) to reduce the burden of denials in evidence-based survivorship care. Due to their negative effects on care delivery and outcomes, utilization management policies, particularly prior authorization, have been advocacy priorities for ASCO for many years (https://old-prod.asco.org/sites/new-www.asco.org/files/content-files/advocacy-and-policy/documents/2020-UM-Update.pdf, https://old-prod.asco.org/sites/new-www.asco.org/files/content-files/advocacy-and-policy/documents/Prior-Auth-Position-Statement.pdf). A recent final rule from the Centers for Medicare & Medicaid Services aimed at streamlining and improving prior authorization in federal health plans is a welcome policy change (https://old-prod.asco.org/news-initiatives/policy-news-analysis/asco-welcomes-new-rule-establishing-electronic-prior.), although its impact on survivorship services remains to be seen. Given our survey findings that commercial insurers were most often the source of a barrier to evidence-based survivorship care, further advocacy will be necessary.

The conclusions of our study are limited to those common diseases where evidence-based survivorship care guidelines exist. As a result, it is unclear how common gaps in coverage exist for less common cancers or for those without clear guidelines for follow-up care. Additionally, the response rate was 22%, which may impact the generalizability of the results. Finally, within this analysis, quantitative assessments were distributed to individuals and practices familiar with survivorship care guidelines. This methodology was intentional; it was felt that the conclusions from this study, where gaps in survivorship care coverage were identified, would be applicable to other programs that may not have as much expertise around survivorship guidelines.

In conclusion, our study demonstrates that gaps in coverage for evidence-based survivorship care services exist, regardless of payer type or region of the country. In an effort to provide quality survivorship care and to reduce the burden on oncology providers and practices in working through denials and prior authorizations, there is a need for better advocacy with payers and improved policy to support quality guideline concordant care for cancer survivors.

### Supplementary Information

Below is the link to the electronic supplementary material.

Supplementary file1 (DOCX 23 KB)

Supplementary file2 (DOCX 14 KB)

## Data Availability

Data are available upon request.
