# Supplementary material for: Coverage for evidence-based cancer survivorship care services
Source: Support Care Cancer. 2024 Feb 17;32(3):165. doi: 10.1007/s00520-024-08359-9 (PMC10874319; doi:10.1007/s00520-024-08359-9)
Supplement: Supplementary file 1 — Supplementary file1 (DOCX 23 KB) [file 520_2024_8359_MOESM1_ESM.docx]

**Supplement One. Survivorship Guidelines in Presence in 2018 at the Time of Survivorship Services Survey Development**

ASCO

- [Management of Chronic Pain in Survivors of Adult Cancers](http://www.asco.org/practice-guidelines/quality-guidelines/guidelines/patient-and-survivor-care#/13021)
- [Breast Cancer Survivorship Care Guideline](http://www.asco.org/practice-guidelines/quality-guidelines/guidelines/patient-and-survivor-care#/9526)
- [Prostate Cancer Survivorship Care Guideline Endorsement](http://www.asco.org/practice-guidelines/quality-guidelines/guidelines/patient-and-survivor-care#/9426)
- [Prevention and Management of Chemotherapy-Induced Peripheral Neuropathy in Survivors of Adult Cancers](http://www.asco.org/practice-guidelines/quality-guidelines/guidelines/patient-and-survivor-care#/9541)
- [Screening, Assessment, and Management of Fatigue in Adult Survivors of Cancer Guideline Adaptation](http://www.asco.org/practice-guidelines/quality-guidelines/guidelines/patient-and-survivor-care#/9546)
- [Screening, Assessment, and Care of Anxiety and Depressive Symptoms in Adults With Cancer Guideline Adaptation](http://www.asco.org/practice-guidelines/quality-guidelines/guidelines/patient-and-survivor-care#/9656)
- [Fertility Preservation in Patients with Cancer](http://www.asco.org/practice-guidelines/quality-guidelines/guidelines/patient-and-survivor-care#/9661)
- [The Integration of Palliative Care into Standard Oncology Care](https://www.asco.org/practice-guidelines/quality-guidelines/guidelines/patient-and-survivor-care#/9671)
- [Prevention and Monitoring of Cardiac Dysfunction in Survivors of Adult Cancer](http://ascopubs.org/doi/pdf/10.1200/JOP.2016.018770)
- [Head and Neck Survivorship Care](http://ascopubs.org/doi/abs/10.1200/JCO.2016.71.8478)
- [Interventions to Address Sexual Problems in People with Cancer Endorsement](https://www.asco.org/practice-guidelines/quality-guidelines/guidelines/patient-and-survivor-care#/28976])
- [Follow-Up Care, Surveillance Protocol, and Secondary Prevention Measures for Survivors of Colorectal Cancer Endorsement](https://www.asco.org/practice-guidelines/quality-guidelines/guidelines/patient-and-survivor-care#/10246)

[NCCN](https://www.nccn.org/professionals/physician_gls/default.aspx#survivorship)

Late Effects/Long-Term Psychosocial and Physical Problems

- Anthracycline-Induced Cardiac Toxicity
- Anxiety and Depression
- Cognitive Function
- Fatigue
- Menopause-Related Symptoms
- Pain
- Sexual Function (female/male)
- Sleep Disorders

Preventive Health

- Healthy Lifestyles
  - Physical Activity
  - Nutrition and Weight Management
  - Supplement Use
- Immunizations and Infections

American Cancer Society

Cancer Survivorship Guidelines

- [For Breast Cancer Survivors](http://www.cancer.org/Healthy/InformationforHealthCareProfessionals/ACSGuidelines/breastcancersurvivorshipguidelines/index.htm)
- [For Colorectal Cancer Survivors](http://www.cancer.org/Healthy/InformationforHealthCareProfessionals/ACSGuidelines/ColorectalCancerSurvivorshipCareGuidelines/index.htm)
- [For Prostate Cancer Survivors](http://www.cancer.org/Healthy/InformationforHealthCareProfessionals/ACSGuidelines/ProstateCancerSurvivorshipCareGuideline/index.htm)
- [For Head and Neck Cancer Survivors](http://www.cancer.org/Healthy/InformationforHealthCareProfessionals/ACSGuidelines/HeadandNeckCancerSurvivorshipCareGuideline/index.htm)

Cancer Care Ontario

Survivorship Evidence-based Series

- [Follow-up Care for Survivors of Lymphoma who have Received Curative-Intent Treatment](https://www.cancercare.on.ca/common/pages/UserFile.aspx?fileId=359493)
- [Follow-up Care and Psychosocial Needs of Survivors of Prostate Cancer](https://www.cancercare.on.ca/common/pages/UserFile.aspx?fileId=342321)
- [Follow-up and Surveillance of Curatively Treated Lung Cancer Patients](https://www.cancercare.on.ca/common/pages/UserFile.aspx?fileId=318621)
- [Models of Care for Cancer Survivorship](https://www.cancercare.on.ca/common/pages/UserFile.aspx?fileId=258056)
- [Follow-up Care, Surveillance Protocol, and Secondary Prevention Measures for Survivors of Colorectal Cancer](https://www.cancercare.on.ca/common/pages/UserFile.aspx?fileId=124839)
