# Supplementary material for: Coverage for evidence-based cancer survivorship care services
Source: Support Care Cancer. 2024 Feb 17;32(3):165. doi: 10.1007/s00520-024-08359-9 (PMC10874319; doi:10.1007/s00520-024-08359-9)
Supplement: Supplementary file 2 — Supplementary file2 (DOCX 14 KB) [file 520_2024_8359_MOESM2_ESM.docx]

**Supplement Two: Qualitative Interview Questions**

1. Close to 1/3 of survey participants indicated denials for maintenance chemotherapy and immunotherapy.

   Can you tell us if this has been a problem at your site?
   If so, can you talk about what the issues you are experiencing?
2. Over half of survey participants indicated issues with denials for supportive care or symptom management services with limited billing codes suggested a potential problem reason (e.g., bone density tests, fatigue assessment/treatment, psychosocial counseling, fertility preservation, physical therapy and occupational therapy, dental evaluations).

   Is this an issue at your site? Are the issues the same across the board for services?
   If not what differences are you experiencing by symptom management service?
   PROBE: IS IT FLAT DENIAL VS NUMBER OF RECOMMENDED ASSESSMENTS/AMOUNT OF SERVICE IS LIMITED?
   ARE COPAYS AN ISSUE?
3. Approximately 1/3 of sites describe CT screening (for new primaries) as an issue for head an neck, gynecologic, Hodgkin lymphoma and lung cancers. Some also describe it as an issue for tests for recurrence for colorectal cancer, gynecologic cancers, and Hodgkin lymphoma.

   Is this an issue at your site?
   What has been your experience?
   PROBE: IS IT FLAT DENIAL VS NUMBER OF TESTS?
4. Of the questions asked, do you know of different coverage issues for neighboring states?
